# Supplementary material for: Association of Mediterranean Diet with Cardiovascular Risk Factors and with Metabolic Syndrome in Subjects with Long COVID: BioICOPER Study
Source: Nutrients. 2025 Feb 12;17(4):656. doi: 10.3390/nu17040656 (PMC11858499; doi:10.3390/nu17040656)
Supplement: Supplementary file 1 [file nutrients-17-00656-s001.zip › nutrients-3462251-supplementary.pdf]

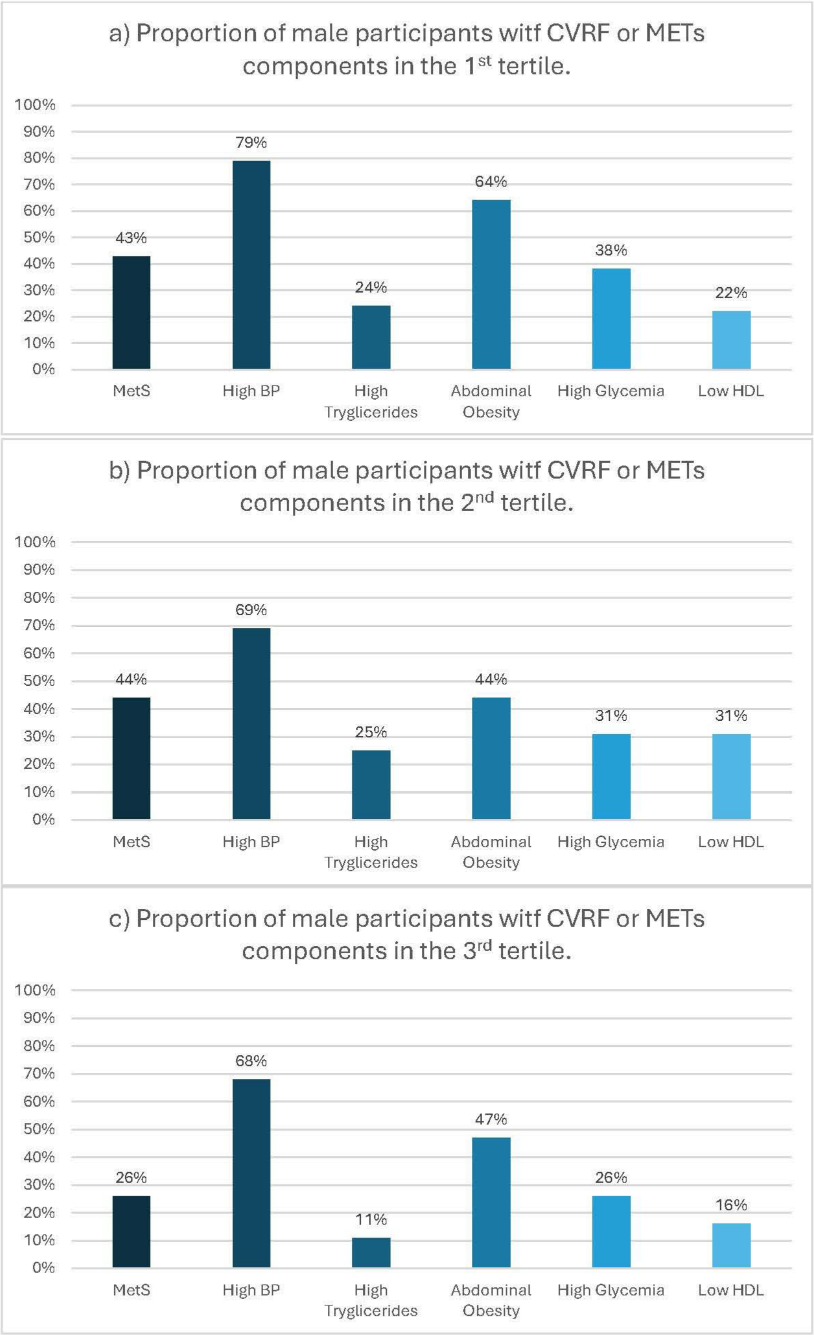

Figure S1. Percentage of MetS and its components across the tertiles of the MD score in men. MetS: metabolic syndrome. CVRF: cardiovascular risk factors. HDL: high-density lipoproteins.

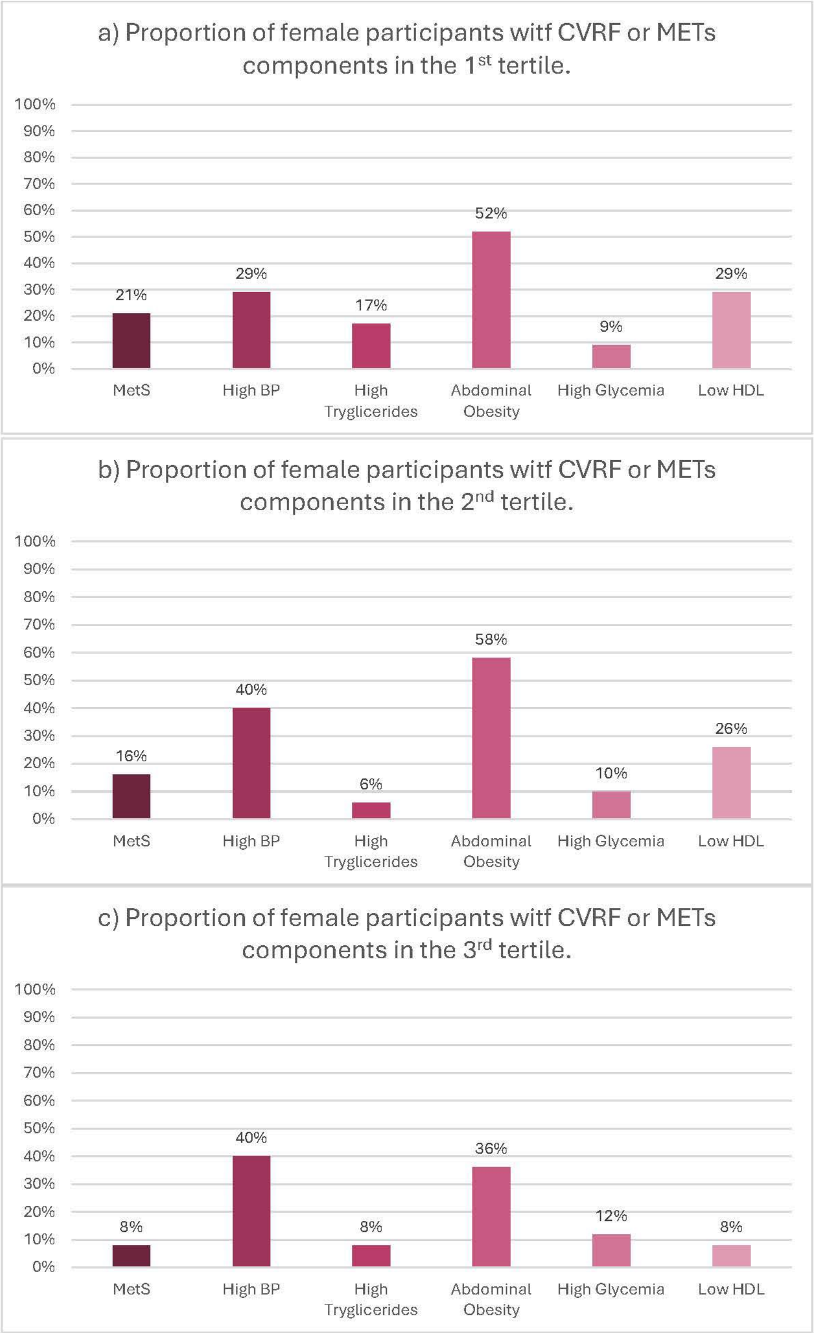

Figure S2. Percentage of MetS and its components across the tertiles of the MD score in women. MetS: metabolic syndrome. CVRF: cardiovascular risk factors. HDL: high-density lipoproteins.
